# Antioxidant properties and antibacterial activity of selected herbal teas

**DOI:** 10.1038/s41598-025-26960-8

**Published:** 2025-11-21

**Authors:** Dariusz Nowak, Lucyna Kłębukowska, Michał Gośliński

**Affiliations:** 1 Department of Nutrition and Dietetics, Faculty of Health Sciences, Ludwik Rydygier Collegium Medicum in Bydgoszcz, Nicolaus Copernicus University in Toruń, Dębowa 3, 85-626 Bydgoszcz, Poland; 2 Department of Food Microbiology, Meat Technology and Chemistry, Faculty of Food Science, University of Warmia and Mazury in Olsztyn, pl. Cieszyński 1, 10-726 Olsztyn, Poland

**Keywords:** Herbal teas, Antioxidant properties, Antibacterial activity, Biochemistry, Drug discovery, Microbiology, Plant sciences

## Abstract

Herbal teas are a valuable source of bioactive compounds, which affect their pro-health properties. Especially polyphenols may play an important role against oxidative stress and could also bring tangible benefits in the treatment of bacterial and viral infections. Nowadays, the consumption of various herbal infusions is becoming more popular. Therefore, the aim of this study has been to evaluate antioxidant and antimicrobial properties of selected herbal teas. The research material was composed of eight herbal infusions: *Cistus incanus* L., *Camellia sinensis* L., *Achillea millefolium* L., *Centaurium erythraea* Rafn, *Leonurus cardiaca* L., *Salvia officinalis* L., *Melissa officinalis* L., *Mentha piperita* L. Antioxidant capacity (DPPH, ABTS), total polyphenol (TP, FBBB) and total flavonoid (TF) content were determined. Furthermore, the antimicrobial activity and the minimal inhibitory concentration (MIC) were evaluated. The results showed the highest antioxidant properties for herbal teas from *Melissa officinalis* L., *Cistus incanus* and *Camellia sinensis*. The lowest values were determined for *Achillea millefolium* and *Centaurium erythraea* teas. All tested herbal infusions presented varied antibacterial activity, although all of them were more efficacious against Gram-positive than Gram-negative bacteria. The highest antimicrobial properties were identified for teas from *Cistus incanus* and *Camellia sinensis*. Only *Melissa officinalis* tea showed activity against some Gram-positive test strains, mainly *S. aureus* and *E. faecalis*. Herbal infusions presented a high polyphenol content but had quite a low total flavonoid content, which suggests that other compounds determine their antioxidant properties, probably numerous phenolic acids, tannins and others. Further studies including the structure of polyphenols should be conducted.

## Introduction

The consumption of herbal beverages is gaining popularity, driven by the fact that many are rich sources of natural bioactive compounds, such as alkaloids, carotenoids, coumarins, flavonoids, polyacetylenes and terpenoids^1^. Like fruits, vegetables and fruit juices, herbal infusions, owing to a number of bioactive compounds they contain, can enhance the body’s antioxidant defences.

Consumption of herbal infusions brings beneficial results in the fight against oxidative stress, responsible for many diseases, and can therefore aid the treatment of bacterial and viral infections. Currently, there is a search for various plant extracts that can bring tangible benefits in the treatment of bacterial and viral infections. *Cistus incanus* and other herbal teas can represent a valuable source of phytochemicals with an interesting wide-spectrum antimicrobial potential.

*Cistus incanus* L. belongs to the Cistaceae family and grows in the Mediterranean regions of southern Europe and North Africa. Dried leaves from *Cistus incan*us are used as herbal tea and dietary supplements^2^. This plant is distinguished by a high content and diverse profile of polyphenolic substances with strong antioxidant activity. *Cistus incanus* contains a number of phytochemicals: flavonoids (myricetin, quercetin, kaempferol and, in smaller amounts, apigenin and naringenin), phenolic acids (gallic acid, coumaric acid, caffeic acid, ferulic acid), tannins, phytosterols, dimeric proanthocyanidins. Various cistus herbal teas have been used in folk medicine for treatment of diarrhoea, fever, and skin disorders. These teas also have antispasmodic, anti-inflammatory and antibacterial properties^3^. It is noteworthy that the European Food Safety Authority (EFSA) has recognized *Cistus incanus* for its health-enhancing natural compounds, which exhibit significant antioxidant properties^2^.

Green tea (*Camellia sinensis* L.) is one of the oldest and most popular beverages in the world. *Camellia sinensis* is grown mainly in Japan, China and Taiwan, and it is classified according to the tradition of green tea leaf processing, the place of origin as well as the type of soil on which the bushes have grown^4,5^. The chemical composition of green tea includes more than ten groups of compounds. The main components are flavonoids (mainly catechins belonging to the flavan-3-ol), with a relatively small content of phenolic acids as well as amino acids, proteins and fats. The number of polyphenolic compounds, including catechins, depends on the climatic and agro-technical cultivation conditions of *Camellia sinensis*. Compared to black tea, green tea has a much higher content of catechins. The higher the catechin content in tea, the higher the antioxidant activity^5,6^. In addition, green tea contains purine alkaloids, such as caffeine (2 –5%), and small amounts of theophylline and theobromine, which are the main contributors to the refreshing effect of tea^6^.

Yarrow (*Achillea millefolium* L.) is an important medicinal plant of the *Asteraceae* family, recognized globally for its therapeutic applicationslower value was determined. The literature indicates the use of *A. millefolium* since ancient times, mainly for various gastrointestinal disorders. Later studies have revealed the anti-inflammatory, antifungal, analgesic, haemostatic, cholagogue, hepatoprotective, and antibacterial effects of this plant^8^. This plant’s anti-inflammatory and antiseptic qualities are attributed to its various bioactive compounds, such as flavonoids (apigenin, luteolin, quercetin, rutin), phenolic acids (caffeic, chlorogenic, salicylic), tannins, terpenes and sesquiterpenes, lactones, betaine, resins and sterols^8^.

*Centaurium erythraea* Rafn (Rafn and Buchs 1800; common centaury) is a plant belonging to the Gentianaceae family, growing in Europe, western Asia and northern Africa. It has also been naturalised in parts of North America, New Zealand, and eastern Australia, where it is an introduced species^9,10^. Extracts from this plant are rich in secoiridoid glycosides, namely gentiopicroside, sweroside, swertiamarin, and in xanthone derivatives^9^. This plant has anti-bacterial, anti-fungal, anti-leishmanial, insecticidal, anti-oxidant, anti-inflammatory, anti-diabetic, and anti-proliferative, as well as gastroprotective, hepatoprotective, dermoprotective, and neuroprotective properties^10,11^.

*Leonurus cardiaca* L. (motherwort) is a perennial herb belonging to the Lamiaceae family, native to Asia and southeastern Europe, with widespread global occurrence in present days^12^. This plant contains furanic diterpenes (labdanes), alkaloids (of special interest being stachydrine), sterols, iridoids, flavonoids, ursolic acid, minerals, and others^12^. The plant was historically used as cardiotonic and for treating gynaecological afflictions. Currently, the composition of *Leonurus cardiaca* L. predisposes it to cardioprotective, antioxidant, antimicrobial, anti-inflammatory, analgesic, nephroprotective, and antiviral properties, among others^12^.

Sage (*Salvia officinalis* L.), which belongs to the family Lamiaceae, is native to the Middle East and Mediterranean areas. *S. officinalis* has a long history of use in preparation of many foods and traditional medicinal preparations in Asia, Latin America and Europe. In recent years, many studies have been conducted to document the traditional uses of *S. officinalis* and to find new biological effects of this plant^13^. Various studies have revealed a wide range of pharmacological activities including anticancer, anti-inflammatory, antibacterial, antioxidant, antimutagenic, antidementia, hypoglycemic, and hypolipidemic effects^13^. This is due to its content of a number of bioactive compounds. The major phytochemicals in *Salvia officinalis* include alkaloids, carbohydrate, fatty acids, glycosidic derivatives, phenolic compounds, polyacetylenes, steroids, terpenes and waxes. Phenolic compounds are represented by flavonoids (quercetin, rutin, epicatechin, epigallocatechin gallate, luteolin-7-glucoside), phenolic acids (caffeic acid, 3-caffeoylquinic acid, chlorogenic acid, ellagic acid, rosmarinic acid), coumarins, and tannins^13,14^.

Lemon balm (*Melissa officinalis* L.) is an edible medicinal herb belonging to the mint family Lamiaceae and the subfamily Nepetoideae. It commonly grows in the Mediterranean region and Western Asia, and it is intensively cultivated in Europe^15,16^. *Melissa officinalis* is a medicinal plant rich in biologically active compounds, such as flavonoids, terpenoids, phenolic acids, tannins, and essential oil^16^. This plant has antioxidant, antimicrobial, cytotoxic and many other pro-health properties^16^.

*Mentha piperita* L. (also known as peppermint) is a genus of plants in the taxonomic family Lamiaceae (mint family), and it is widely distributed around the world. Peppermint contains a variety of ingredients that are classified as peppermint essential oil, and non-essential components including steroids, flavonoids, triterpenoids, phenolic acids, etc.^17^. *Mentha piperita* L. is commonly applied in medications for flu, headache, red eyes, fever and sore throat, and it is also used as a flavouring agent and functional tea^17^.

The herbal plants discussed above are often consumed in the form of tea infusions, and the variety of bioactive compounds they contain means that they will have different antioxidant and antimicrobial properties, which are important to know. Therefore, the aim of the work is to analyse the antioxidant and antibacterial properties of various herbal infusions.

## Materials and methods

### Materials

Eight herbal teas (*Cistus incanus* L., *Camellia sinesis* L., *Achillea millefolium* L., *Centaurium erythraea* Rafn, *Leonurus cardiaca* L., *Salvia officinalis* L., *Melissa officinalis* L., *Mentha piperita* L.) were selected for analyses. All herbal teas had an EU organic farming certificate and were purchased from a specialized tea store.

### Preparation of water infusions

Water infusions were prepared by a modified Bernacka et al.^18^ method. Infusions were prepared in duplicate. First, freshly boiled distilled water (200 mL) was added to dried plant material (2 g). *Camellia sinensis* L., *Salvia officinalis* L., *Melissa officinalis* L. and *Mentha piperita* L. were herbal teas made from leaves, while *Cistus incanus* L., *Achillea millefolium* L., *Centaurium erythraea* Rafn and *Leonurus cardiaca* L. were teas made from whole herbs. The infusions were brewed under cover and after 15 min strained through a fine mesh strainer and filtered through Whatman No. 4 filter paper.

## Methods

The pH of the tested samples was measured with a glass electrode (Hanna Instruments, Olsztyn, Poland) at room temperature.

### Antioxidant capacity

#### DPPH assay

The antioxidant capacity of the extracts was determined by a modified Yen and Chen method, using 0.1 mmol/L methanol solution of 1,1-diphenyl-2-picrylhydrazyl (DPPH, Sigma-Aldrich, St. Louis, MO, USA)^19^. This method is widely used to test the antioxidant capacity of fruit, vegetables and juices. Advantages of the DPPH assay were previously described^20–22^. The procedure was as follows: an amount of 0.1 mL of a sample was added to 2.9 mL of DPPH solution and mixed. The absorbance was measured on a Rayleigh UV-1800 V/VIS spectrophotometer at 517 nm after 30 min of incubation at room temperature in the dark. For each tea extract, samples were analysed in three replicates and the results were used to calculate an average value. The percentage of DPPH scavenging was calculated using Eq. (1):

1 $$\% {\text{ scavenging}} = \left[ {\left( {{\text{A}}_{{{\text{DPPH}}}} {-}{\text{A}}_{{{\text{extract}}}} } \right)/{\text{A}}_{{{\text{DPPH}}}} } \right] \, \times { 1}00$$

where A_DPPH_ is the absorbance of the DPPH blank solution and A_extract_ is the absorbance of the sample solution

The resultant value was then substituted into an equation of a previously prepared 6-hydroxy-2,5,7,8-tetramethylchromane-2-carboxylic acid (Trolox-Sigma-Aldrich) calibration curve. The antioxidant capacity of the samples was expressed as milligrams of Trolox equivalents (Sigma-Aldrich) per 100 mL of sample (mg Tx/100 mL).

#### ABTS assay

The antioxidant capacity was determined by the Re et al.^23^ method with small modifications. In the ABTS method, 2,2’-azinobis-(3-ethyl-benzothiazoline-6-sulfonic acid) diammonium salt (ABTS, Sigma-Aldrich, St. Louis, MO, USA) and potassium persulfate solutions were mixed and stored overnight at room temperature in the dark for 12–16 h. ABTS solution was diluted with methanol to an absorbance of 0.70 ± 0.02 at 734 nm.

After addition of 1.0 mL of diluted ABTS solution (A734 nm = 0.700 ± 0.020) to 0.01 mL of antioxidant compounds or Trolox standards in methanol, the absorbance was measured on a Rayleigh UV-1800 V/VIS spectrophotometer at 734 nm against methanol after 1 min. Quantification was performed using a Trolox standard curve. The antioxidant capacity of the samples was expressed as milligrams of Trolox equivalents (Sigma-Aldrich) per 100 mL of sample (mg Tx/100 mL). The percentage of ABTS scavenging was calculated using Eq. (2):

2 $$\% \;{\text{scavenging}} = \left[ {\left( {{\text{A}}_{{{\text{ABTS}}}} {-}{\text{A}}_{{{\text{extract}}}} } \right)/{\text{A}}_{{{\text{ABTS}}}} } \right] \times {1}00$$

where A_ABTS_ is the absorbance of the ABTS blank solution and A_extract_ is the absorbance of the sample solution.

The resultant value was then substituted into an equation of a previously prepared 6-hydroxy-2,5,7,8-tetramethylchromane-2-carboxylic acid (Trolox-Sigma-Aldrich) calibration curve.

### Phenolic compounds

#### Total polyphenol content

The total polyphenol content (TP) of the samples was determined in the Folin–Ciocalteu assay (Sigma-Aldrich)^24^. First, 0.3 mL of a sample was placed in a 10-mL capacity tube, next 0.05 mL 2 mol/L Folin–Ciocalteu reagent (Sigma-Aldrich, St. Louis, MO, USA) and 0.5 mL 20% sodium carbonate solution were added. The mixture was diluted by addition of 4.15 mL distilled water and mixed. The absorbance was measured on a Rayleigh UV-1800 V/VIS spectrophotometer at 765 nm after 30 min incubation in the dark at room temperature. A calibration curve was performed with gallic acid. The results were expressed as milligrams of gallic acid equivalents per 100 mL of sample (mg GAE/100 mL).

#### Fast Blue BB assay

Fast Blue BB is a novel method described by Medina^25^ to quantify the phenolic compounds through direct interaction of polyphenols with the Fast Blue BB (FBBB) reagent (4-benzoylamino-2,5-diethoxybenzenediazonium chloride hemi(zinc chloride) salt; Sigma-Aldrich) in an alkaline medium. This method demonstrates higher values of gallic acid equivalents (GAE) than the Folin–Ciocalteu assay does^25,26^. An 0.2 mL aliquot of 0.1% Fast Blue BB reagent was added to 2 mL of samples and mixed for 1 min and 0.2 mL 5% sodium hydroxide was added. The absorbance was measured on a Rayleigh UV-1800 V/VIS spectrophotometer at 420 nm after 90 min of incubation in the dark at room temperature. The results are expressed as gallic acid equivalents per 100 mL of sample (mg GAE/100 mL).

#### Total flavonoid content

The total flavonoid content was measured using the colorimetric assay developed by Kapci et al.^21^. Briefly, 0.3 mL of 5% sodium nitrite was added to 1 mL of sample at zero time. After 5 min, 0.3 mL of 10% aluminium chloride was added. At the 6th min, 2 mL of 1 M sodium hydroxide was added. The mixture was diluted by addition of 2.4 mL distilled water and mixed. The absorbance was measured on a Rayleigh UV-1800 V/VIS spectrophotometer at 510 nm. The total flavonoids content was determined by a ( +)-catechin (Sigma-Aldrich) standard curve and was expressed as milligrams of catechin equivalents per 100 mL of sample (mg CAE/100 mL).

### Antibacterial activity of herbal teas

The antibacterial activity in all the analysed herbal teas was determined with the agar well diffusion method according to previous studies^27^. The test strains originated from a collection of strains maintained at the Department of Food Microbiology, Meat Technology and Chemistry of the University of Warmia and Mazury in Olsztyn. The same test strains were used as in studies on the activity of selected juices^27^. Surface cultures (10^5^ CFU/mL) of the test strains were started on sterile Petri plates filled with 20 mL Mueler–Hinton agar medium (Merck). Next, wells of 10 mm size diameter were made with sterile cork borer into agar plates containing the bacterial inoculum and filled with the analysed herbal teas, each in an amount of 0.7 mL. The plates were incubated at optimal temperature (30 °C or 37 °C) for 24–48 h. In the case of the *Clostridium perfringens* strain, incubations were carried in anaerobic conditions After incubation, the antibacterial activity was evaluated by measuring the width of the zone of inhibition (clear) of growth against the indicator organisms in comparison to a control of reference standards. The presence of a growth inhibition zone, regardless of its size, was considered evidence of the antibacterial activity of the tested infusions against the reference strains. All tests were performed in triplicate.

#### Minimal inhibitory concentration (MIC)

For herbal teas that showed antibacterial activity against the tested Gram-negative and Gram-positive strains, the minimal inhibitory concentration (MIC) in relation to the test strains were determined. MIC were determined using the Mueller–Hinton broth dilution method (Merck) using microplates. Bacterial cultures were grown in nutrient broth, and their density was compared to a 0.5 McFarland solution. One hundred μl of bacterial suspension was added to each well. A negative control was prepared using the medium and the test tea, and a positive control was prepared using the medium and bacterial culture^27–29^. The MIC values were expressed as % (v/v).

#### Test strains used in the study

The following strains were submitted to tests:

Gram-positive: *Staphylococcus aureus* G3, *Staphylococcus aureus* 2G, *Staphylococcus aureus* 01, *Enterococcus faecalis* 24, *Enterococcus faecalis* 11, *Enterococcus faecalis* 07, *Listeria monocytogenes* 67, *Listeria monocytogenes* 74, *Listeria inocua* LI0001, *Bacillus cereus* 1, *Bacillus cereus* 9, *Clostridium perfringens* Clpe0001.

Gram-negative: *Escherichia coli* 31, *Escherichia coli* 22, *Escherichia coli* 26, *Escherichia coli* 34, *Klebsiella pneumoniae * 003, *Salmonella typhimurium* 63 s, *Salmonella typhimurium* 235, *Salmonella enteritidis* 61 s, *Pseudomonas aeruginosa* PA0001, *Pseudomonas fluorescens* ATCC13625.

### Statistical analysis

The results were statistically analysed by calculating the mean and standard deviation. The interpretation of the results was performed with MS Excel Analysis ToolPak software (Microsoft, Redmond, WS, USA) applying one-way analysis of variance (ANOVA) and the Tukey’s post hoc test: different letters in the same row or column in the tables indicate statistical significance (at least *p* ≤ 0.05).

## Results and discussion

All the analysed herbal teas had pH between 6.9 and 7.4 (Table 1). The lowest pH values were determined in *Cistus incanus* herbal tea (6.9), followed by *Camellia sinensis* herbal tea (7.0). The highest pH values noted in our study (7.4) were determined in *Centaurium erythraea* Rafn and *Leonurus cardiaca* herbal tea.

**Table 1:** Antioxidant properties of the analysed herbal teas.

	TP (mg GAE/100 mL)	FBBB (mg GAE/100 mL])	TF (mg CAE/100 mL)	pH
*Cistus incanus*	177.7 ± 3.2^b^	212.0 ± 0.7^a^	12.8 ± 0.1^c^	6.9^b^
*Camellia sinensis*	65.3 ± 0.3^c^	199.3 ± 3.5^a^	8.4 ± 0.2^d^	7.0^b^
*Achillea millefolium*	7.6 ± 1.1^e^	7.7 ± 0.2^d^	5.6 ± 0.2^d^	7.3^a^
*Centaurium erythraea*	1.2 ± 0.1f.	1.3 ± 0.1^e^	0.2 ± 0.1^e^	7.4^a^
*Leonurus cardiaca*	32.1 ± 2.1^d^	10.2 ± 0.2^d^	18.6 ± 0.2^c^	7.4^a^
*Salvia officinalis*	84.7 ± 2.9^c^	45.3 ± 0.8^c^	47.0 ± 0.8^b^	7.3^a^
*Melissa officinalis*	252.3 ± 7.4^a^	173.2 ± 1.6^b^	177.8 ± 2.5^a^	7.2^ab^
*Mentha piperita*	67.5 ± 3.4^c^	34.8 ± 0.9^c^	41.0 ± 1.4^b^	7.3^a^

*TP* Total polyphenol content; *FBBB* Fast Blue BB reagent; *TF* Total flavonoid content.

Statistical analysis was performed by one-way ANOVA using the Tukey’s post hoc test: different letters in the same column indicate statistical significance (*p* ≤ 0.05).

**Fig. 1:**
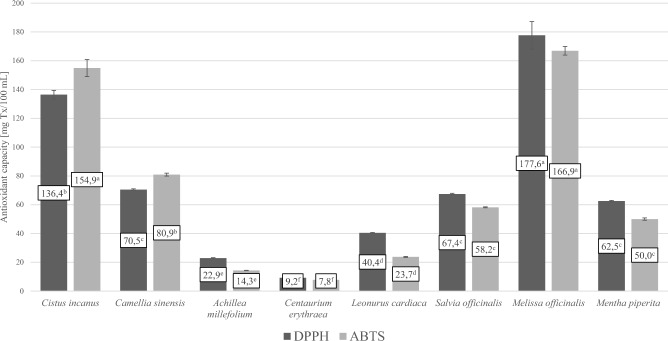
Antioxidant capacity of the analysed herbal teas. *DPPH*, Antioxidant capacity; *ABTS* antioxidant capacity. Statistical analysis was performed by one-way ANOVA using the Tukey’s post hoc test: different letters in data columns (DPPH or ABTS) indicate statistical significance (*p* ≤ 0.05).

Next, the antioxidant properties of all the tested herbal teas were determined (Table 1, Fig. 1). The antioxidant capacity (DPPH assay) was the highest for lemon balm (*Melissa officinalis* L.) herbal tea (177.6 ± 9.6 mg Tx/100 mL). A statistically significantly (*p* ≤ 0.05) lower value was determined for *Cistus incanus* herbal tea (136.4 ± 3.0 mg Tx/100 mL). Even lower antioxidant capacities (from 62.5 ± 0.5 to 70.5 ± 0.5 mg Tx/100 mL) were found in *Camellia sinensis*, *Salvia officinalis* and *Mentha piperita* teas. *Achillea millefolium* and *Centaurium erythraea* teas had the lowest antioxidant capacity, 22.9 ± 0.3 and 9.2 ± 0.1 mg Tx/100 mL, respectively. The antioxidant capacity measured with the ABTS method confirmed the results obtained with the DPPH method. In this assay, we also found the highest antioxidant capacity of teas from *Melissa officinalis* and *Cistus incanus*, 166.9 ± 3.0 and 154.9 ± 5.9 mg Tx/100 mL respectively. In the ABTS method, the lowest values were obtained by *Centaurium erythraea*, *Achillea millefolium* and *Leonurus cardiaca* teas (from 7.8 ± 0.1 to 23.7 ± 0.4 mg Tx/100 mL).

Herbal teas with the highest antioxidant capacities (determined in DPPH and ABTS assays) had the highest content of total polyphenols determined by the Folin-Ciocalteu method (Table 1). *Melissa officinalis* herbal tea had statistically significantly the highest total polyphenol content (252.3 ± 7.4 mg GAE/100 mL). *Cistus incanus* tea also had a relatively high polyphenol content (177.7 ± 3.2 mg GAE/100 mL). *Salvia officinalis*, *Mentha piperita* and *Camellia sinensis* teas had a lower polyphenol content (Table 1). On the other hand, in the second method used to determine the total polyphenol content (FBBB assay), teas from *Cistus incanus* and *Camellia sinensis* gave the highest values*. Melissa officinalis* had a slightly lower polyphenol content than teas from *Cistus incanus* and green tea. In this stage of the study, as with the DPPH method, the lowest amounts of polyphenols were determined in teas from *Centaurium erythraea* and *Achillea millefolium*—1.3 ± 0.1 and 7.7 ± 0.2 mg GAE/100 mL. In our study, *Melissa officinalis* tea had the highest total flavonoid content of all the teas analysed (Table 1). This coincided with the results mentioned above, in which we reported the highest antioxidant capacity and total polyphenol content of lemon balm tea. Teas from *Salvia officinalis* and *Mentha piperita* had a relatively high total flavonoid content. The other herbal teas had more non-flavonoid polyphenols.

The high antioxidant properties of lemon balm (*Melissa officinalis*) were also pointed out by other researchers. Gorjanović et al.^30^ found the highest total polyphenol content in lemon balm tea and black tea, and a low content in yarrow tea. In that study, green tea had a slightly higher total polyphenol content than in our study (81.9 vs. 65.3 mg GAE/100 mL). Furthermore, lemon balm tea had a higher total polyphenol content than green tea^30^, which was in line with our results. In addition to lemon balm tea, we showed that *Cistus incanus* tea also had high antioxidant properties. It had high antioxidant capacity and high total polyphenol content. Many studies have reported that aqueous infusions prepared from *Cistus incanus* leaves are considered to be a source of polyphenols, not only flavonoids, but also proanthocyanidins, ellagitannins and phenolic acid^31,32^. This would explain a rather high content of non-flavonoid polyphenols in *Cistus incanus* infusions. This is also pointed out by Bernacka et al.^18^, who detected a rather high content of non-flavonoid polyphenols (TPC-TF) in analysed *Cistus incanus* infusions. In that study, the total polyphenol content of infusions of *Cistus incanus* from Greece, Albania, or Turkey was similar^18^. However, in another study, the different *Cistus incanus* extracts from Greece, Turkey and Albania analysed had different values for the total polyphenol content (range 14–623 mg/g d.w.), with *Cistus incanus* extracts from Greece achieving the highest values^33^. We also observed high polyphenol content (in the FBBB assay) in *Camellia sinsnesis* tea and *Salvia officinalis* tea (in the Folin-Ciocalteu TP assay). Evidence from several studies suggests that *Salvia officinalis* has strong antioxidant properties and counteracts oxidative stress^13^, although the results of the total polyphenol content and antioxidant capacity were lower than lemon balm and green tea^30^. We found the lowest antioxidant properties (lowest total polyphenol content and lowest antioxidant capacity) in tea from *Centaurium erythraea.* The low antioxidant capacity of the *Centaurium erythraea* is confirmed by other studies. Šiler et al.^34^ showed that secoiridoides contained in *C. erythraea* lack antioxidant activity. Xanthones may be responsible for the minor antioxidant properties of this plant. These compounds have hydroxyl groups in their structure that can easily provide electrons and quench the DPPH radical^9^. In our work, the total polyphenol content and antioxidant capacity of *Achillea millefolium* tea was also relatively low compared to other herbal teas. In the study by Dias et al.^35^, the total polyphenol content and total flavonoid content in *Achillea millefolium* infusions were higher. In contrast, Salomon et al.^36^ found significantly lower antioxidant properties of *Achillea millefolium* compared to *Achillea atrata*.

Our study showed a high correlation between total polyphenol content (the Folin-Ciocalteu method) and antioxidant capacity in both the DPPH (0.994) and ABTS (0.967) assays (Table 2). Gorjanović et al.^30^ showed a lower correlation of 0.811 and 0.618, respectively. We determined a high correlation between DPPH and ABTS assays (0.981), while Gorjanović et al.^30^ obtained a relatively low correlation (0.690). Another study found a strong positive correlation between DPPH and TP (~ 0.99) and moderate correlation with FBBB, TF (~ 0.8). Furthermore, these studies showed a high correlation between DPPH and ABTS (~ 0.98)^33^.

**Table 2:** The correlation coefficients (R^2^).

	DPPH	ABTS	TP	FBBB	TF
DPPH	1				
ABTS	0.981	1			
TP	0.994	0.967	1		
FBBB	0.797	0.883	0.748	1	
TF	0.756	0.635	0.792	0.327	1

*DPPH* Antioxidant capacity; *ABTS* Antioxidant capacity; *TP* Total polyphenol content; *FBBB* Fast Blue BB reagent; *TF* Total flavonoid content.

We have also seen a moderate correlation between the TP and FBBB methods (0.748). This was due the differences in both methods. In our study we noted that the results on the total polyphenol content was higher according to the FBBB method than obtained with the Folin-Ciocalteu assay, but only in the case of *Cistus incanus* tea and *Camellia sinensis tea*. This was similar to earlier observations, but it was about other products^37–39^. Similar or lower polyphenol content values in the FBBB method in the other teas analyzed can be explained by the presence of other bioactive compounds and differences in the selectivity of these methods. The FBBB method, developed by Medina, is less popular than the TP method, but it is more specific to phenols and less affected by other interfering compounds. Major disadvantage of the Folin-Ciocalteu assay is that the reagent reacts with reducing substances and measures the total reducing capacity of a sample rather than of the phenolic compounds^37–39^. It has been shown that not only phenols, but also other compounds (e.g. certain vitamins, sugars, proteins, and thiols) can react with the Folin-Ciocalteu reagent, confirming the weaker specificity of this method^40^. Both methods (TP and FBBB) were further investigated for their susceptibility to interference in different (plant) matrices. Folin–Ciocalteu assay showed interferences for 75% of the flours (attributed to reducing sugars and enediols), whereas FBBB only for legumes and nuts (attributed to the presence of tyrosine). Both methods presented excellent reproducibility, but FBBB displayed 1.5 times higher sensitivity^41^. Despite its known interferences, Folin–Ciocalteu assay is still widely employed in different matrices^42^. Recently, a high correlation between the FBBB method for determining polyphenols and the HPLC–UV method in olive oil has also been demonstrated^43^.

### Antibacterial activity of herbal teas

The analysed infusions demonstrated varied antimicrobial activity. Such differences are also indicated by the findings achieved by other researchers, who investigated the antimicrobial activity of various herbal infusions, including aqueous, ethanol and methanol ones^44,45^. In our experiment, we tested aqueous extracts (teas) from common herbs: mint (*Mentha piperita*), sage (*Salvia officinalis*), lemon balm (*Melisa officinalis*) and green tea (*Camelia sinensis*), as well as well less popular ones, but all with pro-health potential, such as cistus (*Cistus incanus*), yarrow (*Achillea millefolium*), common centuary (*Centaurium erythraea* Rafn) and motherwort (*Leonurus cardiaca*). The results of the antimicrobial activity determined in the tested infusions equivocally indicate that two of the herbs did not have such an effect on any of the tested bacterial strains. These were infusions from yarrow (*Achillea millefolium*) and common centuary (*Centaurium erythraea*) (Tables 3 and 4). Also, the infusion from motherwort (*Leonurus cardiaca*) showed very low antimicrobial potential. This means that 25% of the tested infusions did not demonstrate any antimicrobial potential. In their work, Hacioglu et al.^45^ revealed a lack of antimicrobial activity of some of the teas they tested. More specifically, out of 31 herbal infusions submitted to tests, 15 were shown to demonstrate antimicrobial activity, meaning that 52% of the tested herbal teas did not inhibit the growth of the tested microbial strains.

**Table 3:** Antimicrobial activity of herbal teas against Gram-positive strains.

Tested herbal teas	Zone of inhibition [mm]											
	Gram-positive test strains											
	*Staphylococcus aureus*			*Enterococcus faecalis*			*Listeria monocytogenes*		*Listeria inocua*	*Bacillus cereus*		*Clostridium perfringens*
	*G3*	*2G*	*01*	*24*	*11*	*07*	*67*	*74*	*LI0001*	*1*	*9*	*Clpe0001*
*Cistus incanus*	24 ± 0.4^c^	32 ± 1.0^b^	22 ± 0.2^c^	0	0	20 ± 1.8^b^	18 ± 0.4^b^	26 ± 1.2^a^	0	14 ± 0.4^b^	20 ± 1.0^b^	0
*Camellia sinensis*	30 ± 0.9^b^	36 ± 1.8^a^	30 ± 0.0^a^	0	0	28 ± 2.4^a^	21 ± 0.8^a^	26 ± 0.5^a^	0	20 ± 0.8^a^	22 ± 2.1^b^	0
*Achillea millefolium*	0	0	0	0	0	0	0	0	0	0	0	0
*Centaurium erythraea*	0	0	0	0	0	0	0	0	0	0	0	0
*Leonurus cardiaca*	0	25 ± 0.6^d^	0	0	0	0	0	0	0	0	0	0
*Salvia officinalis*	24 ± 0.2^c^	24 ± 0.4^d^	0	0	0	0	0	0	0	0	0	0
*Melissa officinalis*	35 ± 1.2^a^	28 ± 0.2^c^	31 ± 2.6^a^	0	22 ± 0.3^b^	15 ± 1.6^c^	0	0	0	0	26 ± 1.4^a^	0
*Mentha piperita*	26 ± 0.4^c^	30 ± 0.9^b^	26 ± 0.8^b^	0	35 ± 1.2^a^	0	0	0	0	0	0	0

Statistical analysis was performed by one-way ANOVA using the Tukey’s post hoc test: different letters in the same column indicate statistical significance (*p* ≤ 0.05).

**Table 4:** Antimicrobial activity of herbal teas against Gram-negative strains.

Tested herbal teas	Zone of inhibition [mm]									
	Gram-negative test strains									
	*Escherichia coli*				*Klebsiella pneumoniae*	*Salmonella* typhimurium		*Salmonella* enteritidis	*Pseudomonas aeruginosa*	*Pseudomonas fluorescens*
	*31*	*22*	*26*	*34*	*003*	*63 s*	*235*	*61 s*	*PA0001*	*ATCC13625*
*Cistus incanus*	0	14 ± 0.9^a^	20 ± 2.2^a^	26 ± 1.4^a^	16 ± 1.6^a^	19 ± 2.4^a^	22 ± 2.8^a^	0	17 ± 0.0^b^	16 ± 2.4^a^
*Camellia sinensis*	0	16 ± 0.5^a^	21 ± 1.8^a^	20 ± 2.2^b^	19 ± 1.9^a^	20 ± 0.9^a^	20 ± 1.2^a^	0	19 ± 1.2^a^	19 ± 0.9^a^
*Achillea millefolium*	0	0	0	0	0	0	0	0	0	0
*Centaurium erythraea*	0	0	0	0	0	0	0	0	0	0
*Leonurus cardiaca*	0	0	0	0	0	0	0	0	0	0
*Salvia officinalis*	0	0	0	0	0	0	0	0	0	0
*Melissa officinalis*	0	0	0	0	0	0	0	0	0	0
*Mentha piperita*	0	0	0	0	0	0	0	0	0	0

Statistical analysis was performed by one-way ANOVA using the Tukey’s post hoc test: different letters in the same column indicate statistical significance (*p*≤ 0.05).

The available research papers dealing with the antimicrobial activity of yarrow (*Achillea millefolium*) deliver the findings that confirm it, unlike our results. However, the microbial activity of this herb was not analysed in aqueous infusions. Grigore et al.^46^ proved the antimicrobial activity against the Gram-positive bacteria (*S. aureus*), Gram-negative bacteria (including *Escherichia coli*, *Klebsiella pneumoniae*, *Pseudomonas aeruginosa*) and fungi, including *Candida albicans*, produced by different extracts of yarrow (*Achillea millefolium*), such as hydroalcoholic, hexane, and methanol ones. Similar extracts were studied by Karaalp et al.^47^, who demonstrated the antimicrobial activity of the analysed yarrow (*Achillea millefolium*) extracts against Gram-negative and Gram-positive bacteria, where the effect on Gram-positive bacteria (*Bacillus cereus*, *Enterococcus faecalis* and *Staphylococcus aureus*) was stronger than on *Escherichia coli*. A slightly stronger activity of the analysed infusions against strains of Gram-positive bacteria was also demonstrated in our study (Table 3). The growth of only two strains from this group, *Listeria inocua* L1001 and *Clostridium perfringens* Clpe0001, was not inhibited by any of the infusions submitted to our study. As for Gram-negative bacteria, only two infusions, from cistus (*Cistus incanus*) and green tea (*Camelia sinensis*), showed antimicrobial activity against most of the tested strains (Table 4). In this group of bacteria, similarly to Gram-positive bacteria, none of the infusions inhibited the growth of two tested strains. These were *Escherichia coli* 31 and *Salmonella* Enteritidis. The other strains of the genus *Escherichia coli* and the remaining serotypes of *Salmonella* sp. rods were inhibited by the mentioned infusions. These results substantiate the assumption that the sensitivity of bacteria to herbal infusions, same as to other environmental factors, is a strain-related trait. When analysing the research results, we could conclude that infusions of lemon balm (*Melisa officinalis*) and peppermint (*Mentha piperita*) showed antimicrobial activity only towards Gram-positive bacteria, and a much higher activity was noted against spherical bacteria (Table 3). Infusions from peppermint (*Mentha piperita*) and lemon balm (*Melisa officinalis*) inhibited the growth of all tested strains of *S. aureus* and some strains of *E. faecalis*. Additionally, in comparison to *Mentha piperita*, lemon balm tea inhibited the growth of one of the two tested strains of *Bacillus cereus.* In turn, tea from sage (*Salvia officinalis*) showed inhibitory activity against two of the twelve Gram-positive strains put to tests (Table 3).

Abdei-Naime et al.^15^ demonstrated the inhibitory effect of *Melisa officinalis* on strains *Staphylococcus aureus* and *Pseudomonas aeruginosa*, whereas no antimicrobial effect on *Escherichia coli* and *Klebsiella pneumoniae* was confirmed^15^*.* These results are comparable to our findings, except the Gram-negative bacteria *Pseudomonas aeruginosa*, which were not affected by *Melissa officinalis* infusion. Several authors pointed that *Melissa officinalis* is a plant which is rich in polyphenolic compounds, but the extraction method and the solvent used play a significant role^48,49^.

In a review paper dedicated to the antimicrobial activity of lemon balm (*Melisa officinalis*) and the plant’s potential as a food preservative, Carvalho et al.^50^ cite numerous examples of the antimicrobial effects of this herb against bacteria, fungi and viruses. However, it needs to be mentioned that essential oils demonstrate a much higher activity than other plant extracts. When analysing the research results quoted in the above article^50^, it emerges that particular herbs show highly varied antimicrobial effects against tested strains of microbes, which has also been noted in our study. Recent studies have shown that this plant, through various mechanisms, can also fight viruses, including Severe Acute Respiratory Syndrome Coronavirus 2 (SARS-CoV-2), Herpes Simplex Virus (HSV), and Human Immunodeficiency Virus (HIV)^51^.

As regards *Mentha piperita*, similarly to our findings, Bardaweel et al.^52^ report its moderate antioxidant effect and weak antimicrobial activity. The inhibitory effect of *Mentha piperita* against *S. aureus* has been confirmed by others^53^. In another study, on the antibacterial activity of peppermint oil and different peppermint extracts, Singh et al.^54^ found that the highest inhibitory activity against the indicator strains was demonstrated by essential oils as well as ether, chloroform and acetate extracts of peppermint. Distinctly lower activity was determined in aqueous and ethanol extracts. Same as in our experiment, the cited authors concluded that *S. aureus* was most sensitive to the tested substances, while Gram-negative *E. coli* rods were the least sensitive ones^54^. Interesting results regarding peppermint (*Mentha piperita*) extracts were obtained by Faris Shalayel et al.^55^, who concluded that peppermint extracts showed antimicrobial activity against pathogenic bacteria, including MRSA (Methicillin-resistant *S. aureus*) and MRSE (Methicillin-resistant *S. epidermidis*) strains^55^.

Sage (*Salvia officinalis*) tea was characterized by lower antimicrobial activity than teas made of *Mentha piperita* and *Melisa officinalis*, as it only showed antimicrobial effects on two of the three tested strains of *S. aureus*. Garcia et al.^56^ also demonstrated an effective antimicrobial activity against the strain *S. aureus* and a lack of such an effect on *Streptococcus agalactiae, Candida albicans* and *Candida tropicalis.* The authors concluded that *Salvia officinalis* L. could contribute to the inhibition of pathogens in the skin microbiota^56^.

The infusion from motherwort (*Leonurus cardiaca*) showed antimicrobial activity only towards one tested strain, *S. aureus* 2G (Table 3). Micota et al.^57^ also demonstrated its activity against *S. aureus.*

The minimum inhibitory concentration (MIC) for each infusion was determined to be at a level of 7.5% (v/v) for Gram-positive strains 10% (v/v) for Gram-negative bacteria (Table 5). In this case, also *Camellia sinensis* and *Cistus incanus* teas were the most effective towards a wide range of microorganisms.

**Table 5:** Minimal inhibitory concentration herbal teas (MIC) on tested bacteria strains.

Tested strain	Herbal teas					
	*Cistus incanus*	*Camellia sinensis*	*Leonurus cardiaca*	*Salvia officinalis*	*Melissa officinalis*	*Mentha piperita*
	MIC [% (v/v)]					
*Staphylococcus aureus* G3	7.5^b^	7.5^b^	–	7.5^b^	7.5^b^	7.5^b^
*Staphylococcus aureus* 2G	7.5^b^	7.5^b^	10.0^a^	7.5^b^	7.5^b^	7.5^b^
*Staphylococcus aureus* 01	7.5^b^	7.5^b^	–	–	7.5^b^	7.5^b^
*Enterococcus faecalis* 11	–	–	–	–	7.5^b^	7.5^b^
*Enterococcus faecalis* 07	7.5^b^	7.5^b^	–	–	7.5^b^	–
*Listeria monocytogenes* 67	7.5^b^	7.5^b^	–	–	–	–
*Listeria monocytogenes* 74	7.5^b^	7.5^b^	–	–	–	–
*Bacillus cereus* 1	7.5^b^	7.5^b^	–	–	–	–
*Bacillus cereus* 9	7.5^b^	7.5^b^	–	–	10.0^a^	–
*Escherichia coli* 22	10.0^a^	10.0^a^	–	–	–	–
*Escherichia coli* 26	10.0^a^	10.0^a^	–	–	–	–
*Escherichia coli* 34	10.0^a^	10.0^a^	–	–	–	–
*Klebsiella pneumoniae* 003	10.0^a^	10.0^a^	–	–	–	–
*Salmonella* Typhimurium 63 s	10.0^a^	10.0^a^	–	–	–	–
*Salmonella* Typhimurium 235	10.0^a^	10.0^a^	–	–	–	–
*Pseudomonas aeruginosa* PA0001	10.0^a^	10.0^a^	–	–	–	–
*Pseudomonas fluorescens* ATCC13625	10.0^a^	10.0^a^	–	–	–	–

– Not appointed.

Statistical analysis was performed by one-way ANOVA using the Tukey’s post hoc test: different letters indicate statistical significance (*p*≤ 0.05).

To summarise, it should be underlined that the highest antimicrobial potential against the tested strains was demonstrated by two infusions, of *Cistus incanus* and *Camellia sinensis*. They were the only infusions that inhibited the growth of Gram-negative bacteria, and the increased resistance of Gram-negative, and the greater resistance of Gram-negative bacteria to the analysed infusions could be attributed to the presence of lipopolysaccharides in their outer membrane, which improve the resistance to antimicrobial factors. Furthermore, *Cistus incanus* and *Camellia sinensis* teas, along with *Melissa officinalis* tea, had the highest polyphenol content and the highest antioxidant capacity. These samples also had the highest efficacy against Gram-positive and Gram-negative strains, with the exception of *Melissa officinalis*, which showed inhibitory abilities only against Gram-positive strains, which have a thinner cell wall and are more susceptible to the effects of polyphenols.

The capability of green tea to inhibit food-borne pathogens, including *E. coli*, *Staphylococcus aureus*, *Salmonella typhimurium*, *Listeria monocytogenes* was first indicated by Hamilton-Miller^58^. However, it is unclear which green tea ingredients act as main antibacterial agents, and which bacteria are most strongly inhibited. This gap in knowledge is implicated in a review paper by Zhao et al. ^6^. It has been determined that epigallocatechin gallate (EGCG) has the highest antibacterial activity, exerting the most significant effect on *S. aureus*^6^. However, there is a lack of research focusing on this question. Our study therefore provides a broader view of the antibacterial properties of green tea and other herbal infusions towards as many as 22 Gram-positive and Gram-negative strains.

With respect to cistus (*Cistus incanus*), the antimicrobial activity of its essential oils, which demonstrate high potential, is studied more often^59^. In one of few studies including a commercial hydroalcoholic extract of *Cistus incanus*, it was determined that *Staphylococcus aureus* was more sensitive than *Escherichia coli* to the tested substance^60^. The results obtained in our experiment reveal antimicrobial potential of cistus (*Cistus incanus*) infusions, which was characterised by a high content of polyphenols and high antioxidant capacity.

## Conclusions

The analysis of antioxidant and antibacterial properties of eight herbal teas led to several interesting findings. The highest antimicrobial activity against all tested strains of both Gram-positive and Gram-negative bacteria was determined for *Cistus incanus* and *Camellia sinensis* teas. Apart from these two teas, only *Melissa officinalis* tea showed activity against some Gram-positive test strains, mainly the spherical bacteria *S. aureus* and *E. faecalis*. The high antimicrobial activity of *Cistus incanus*, *Camellia sinensis* and *Melisa officinalis* teas was the result of the rich content of polyphenols (TP and FBBB assays) and the highest antioxidant capacity (DPPH and ABTS assays). It is also worth noting that all the tested herbal teas showed varying antimicrobial activity, but were more effective against the Gram-positive tested strains than against the Gram-negative strains. The teas from *Cistus incanus* and *Camellia sinensis* that showed the highest antimicrobial activity had a high total polyphenol content, but a fairly low total flavonoid content. This suggests that other (non-flavonoid) compounds determine the antioxidant properties of these teas, probably numerous phenolic acids, tannins and others.

We are aware of the limitations of our study. We used herbal infusions to analyse the antimicrobial activity and antioxidant properties. When other solvents and extraction methods are used, the results may be different. However, it is important to note that aqueous infusions are a common form of consuming herbal teas and can provide tangible benefits to human health.

The high antioxidant properties and strong antimicrobial activity of *Cistus incanus*, *Camellia sinensis*, and *Melissa officinalis* teas mean that they can be used in the production of functional beverages with health-promoting properties. The high antimicrobial activity of these teas reduces the necessity of preservatives against bacteria, but their effectiveness against mold requires further research. In addition, the richness of polyphenols in these teas means that they will have a beneficial effect on health due to their antioxidant, anti-inflammatory, immunomodulatory, and antibacterial properties. These makes them an excellent form in prevention against cardiovascular, cancerous, and neurodegenerative diseases. The stability of these beverages, the selection of appropriate packaging, and proper storage conditions require refinement. Further studies with the use of herbal teas, including the structure of polyphenols and other bioactive compounds demonstrating antimicrobial activity, should be conducted.

## Data Availability

The datasets used and/or analyzed during the current study are available from the corresponding author on reasonable request.
